# Digestive Ferments in Urine

**Published:** 1913-11

**Authors:** 


DIGESTIVE FERMENTS IN URINE. Tachau, *Zeit. f. Klin. Med.*, holds that urinary tests for pepsin, etc., cannot be relied on in the diagnosis of digestive conditions.

